# Onion-like carbon unlocks high PdNiO nanocatalyst dispersion for outstanding thermal methane oxidation

**DOI:** 10.1039/d5na01028d

**Published:** 2026-01-21

**Authors:** Ahmed Gamal, Adewale K. Ipadeola, Mostafa H. Sliem, Siham Y. A. Al-Qaradawi, Aboubakr M. Abdullah, Kenneth I. Ozoemena

**Affiliations:** a Center for Advanced Materials, Qatar University Doha 2713 Qatar ak.ipadeola@qu.edu.qa bakr@qu.edu.qa; b Molecular Sciences Institute, School of Chemistry, University of the Witwatersrand Private Bag 3, PO Wits Johannesburg 2050 South Africa kenneth.ozoemena@wits.ac.za; c Department of Chemistry and Earth Sciences, College of Arts and Sciences, Qatar University Doha 2713 Qatar

## Abstract

Methane must undergo complete catalytic oxidation to reduce the emission of unburned methane from power plants and natural gas engines. However, the poor temperature stability of carbon-based supports frequently restricts their usage in methane oxidation. This limitation can be addressed by modifying the carbon structure to enable the development of thermally resilient catalysts. This study utilises onion-like carbon (OLC), a support material made from nanodiamonds by high-temperature calcination, to disperse palladium (Pd) nanoparticles (Pd/OLC). The choice of OLC as the support is based on its distinct physicochemical merits (*i.e.*, enhanced graphitization, a more ordered but defect-rich architecture, better thermal transport and porosity, gas-accessible active sites, improved electrical conductivity and structural stability). The resultant OLC promoted exceptional catalytic activity in the Pd/OLC by offering increased graphitisation, superior gas transport, accessible active sites, and exceptional temperature stability. The effect of adding nickel oxide (NiO) to Pd/OLC in PdNiO/OLC was also investigated, and the results show increased catalytic effectiveness through improved surface area, refined metal dispersion, and reduced particle size. PdNiO/OLC achieves full methane oxidation (*T*_100_) at a lower temperature (400 °C) than Pd/OLC (450 °C) and commercial Pd/C (650 °C). These results demonstrate the potential of OLC as a strong carbon support for gas-phase catalytic processes at high temperatures, which extends beyond methane combustion.

## Abstract 2

Synthesized PdNiO nanocatalysts embedded with onion-like carbon (PdNiO/OLC) enable full methane oxidation at 400 °C, outperforming Pd/OLC and Pd/C due to its enhanced graphitization, high surface area, and optimized metal dispersion.

## Graphical abstract

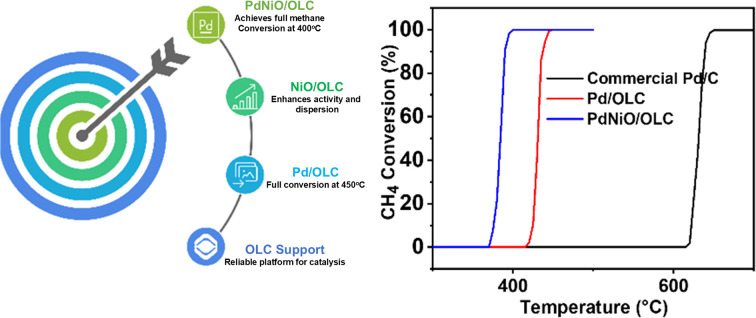

## Introduction

The main component of natural gas, methane (CH_4_), has become an essential energy source because of its high calorific value and widespread availability. It is a key fuel for large-scale power generation systems, home heating, and transportation. However, its effects on the climate are significant, as CH_4_ has a global warming potential 20 times greater than that of carbon dioxide (CO_2_).^1,2^ The atmospheric radiative force and climate stability are disproportionately affected by the emission of even small amounts of uncombusted CH_4_, especially from natural gas leakage and vehicle exhaust. Thus, catalytic methane oxidation has gained significant scientific and technological attention as a means of reducing methane emissions. The high operating temperatures (800–1600 °C) required by conventional thermal combustion processes restrict their economic viability and practical efficiency. The fabrication of sophisticated nanostructured catalysts that permit full methane oxidation (*T*_100_) at significantly lower temperatures has been the focus of much study to overcome these obstacles.^3,4^ These developments enhance the overall energy efficiency of combustion systems while also lowering the greenhouse footprint linked to the usage of natural gas. Competing processes like steam reforming (eqn (1)) and partial oxidation (eqn (2)) can occur during the oxidation process, which can impact the yield and selectivity of the intended products. The primary mechanism for methane abatement across heterogeneous catalytic systems is defined by the full oxidation of methane to carbon dioxide and water (eqn (3)),^5^ which is the major reaction pathway.

1 CH_4_ + H_2_O → CO + 3H_2_

2 CH_4_ + 3/2O_2_ → CO + 2H_2_O

3 CH_4_ + 2O_2_ → CO_2_ + 2H_2_O

Palladium (Pd)-based nanocatalysts have long been regarded as one of the most promising materials for effective methane oxidation due to their remarkable catalytic activity and stability under a range of operating conditions.^6,7^ However, the physicochemical properties of the support material have a significant impact on their overall performance. Because of their robustness and well-established surface interactions, classic metal oxide supports, like SiO_2_ and AlO_3_, have dominated catalyst design for catalytic reactions. In contrast, carbon-based supports have drawn relatively less attention, mostly because of their low temperature resistance in oxidative settings. However, onion-like carbon (OLC) has attracted renewed attention due to recent developments.^8–10^ It features a multilayered, quasi-spherical structure and has physicochemical merits, including increased graphitization, a more ordered yet defect-rich structure, superior thermal transport and gas-accessible active sites.^10,11^ It also offers a large surface area with superior porosity,^12^ improved conductivity and structural stability.^13^ These properties make OLC an incredibly alluring support platform for improving Pd nanoparticle dispersion, activity, and durability in methane oxidation catalysis.

In this work, PdNiO and Pd nanocatalysts embedded on OLC, which are referred to as PdNiO/OLC and Pd/OLC, respectively, are investigated for the thermocatalytic oxidation of methane. This report is the first to study OLC-supported Pd-based nanocatalysts for methane oxidation. The results demonstrate the potential of OLC as a versatile and effective support, expanding its use beyond methane oxidation to include more extensive thermocatalytic application.

## Methodology

### Preparation of PdNiO/OLC and Pd/OLC

Nanodiamond powder was annealed at 1300 °C for 3 h in an inert argon (Ar) environment to synthesize the OLC. After being dispersed in 50 mL of ultrapure water, the resulting OLC (0.37 g) was magnetically stirred for 30 min. Then, aqueous solutions of K_2_PdCl_4_ (0.115 g) and NiCl_2_·6H_2_O (0.152 g) dissolved in 10 mL of ultrapure water were added dropwise to the OLC dispersion, and the mixture was agitated for another 20 min. After bringing the pH of the final mixture down to 12, it was refluxed for 1 h at 80 °C. After centrifugation, the resulting PdNiO/OLC composite was periodically cleaned with water until its pH was neutral and then vacuum-dried for 24 h at 80 °C. The same method was used to prepare a Pd/OLC sample, except that NiCl_2_·6H_2_O was not added. A commercial Pd/C catalyst (Sigma-Aldrich) was used for benchmarking under the same experimental conditions.

### Methane oxidation catalytic tests

**Scheme 1:**
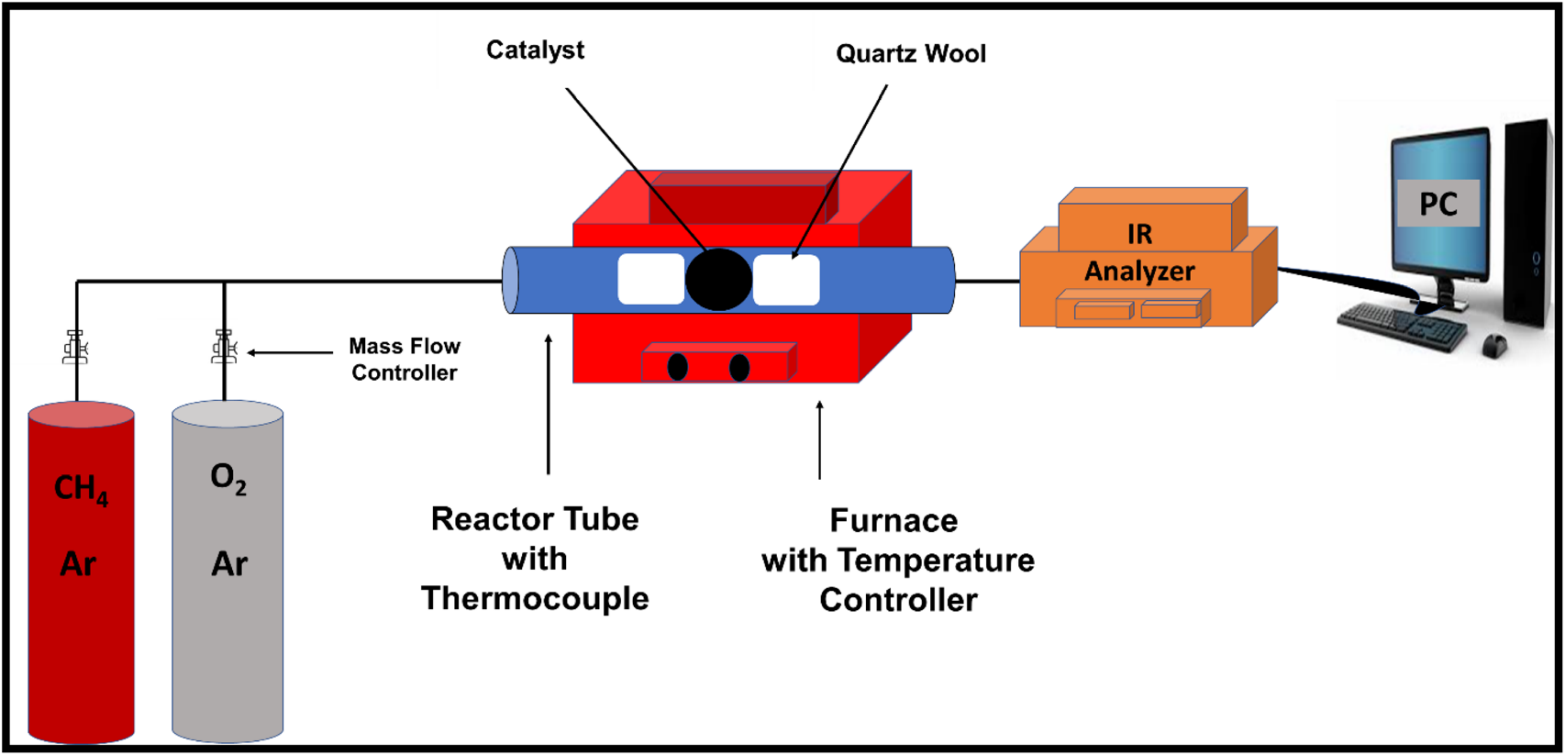
Schematic diagram of the experimental setup used in methane oxidation thermo-catalysis.

Methane oxidation was carried out at atmospheric pressure using the experimental setup shown in Scheme 1. 0.1 g of the as-prepared nanocatalyst was loaded between quartz wool plugs in a fixed-bed reactor for each run, and a feed gas, consisting of 1 vol% CH_4_, 20 vol% O_2_ and balanced with Ar, was passed through at a total flow rate of 20 mL/min. The samples were activated in 20 vol% O_2_/Ar at 500 °C for 1 h before being cooled to room temperature in preparation for catalytic testing. Subsequently, the reaction temperature was raised from ambient temperature (25 °C) to 650 °C. A Yokogawa IR200 infrared spectrometer was used to measure the effluent gases after they passed through a condenser to eliminate moisture. The IR200 was used because of its rapid, continuous monitoring, and excellent accuracy. Additionally, its output is typically gas concentration (*i.e.*, CH_4_ conversion (%) *versus* time or temperature). The percentage methane conversion and turnover frequency (TOF) were calculated using eqn (4) and (5), respectively.4
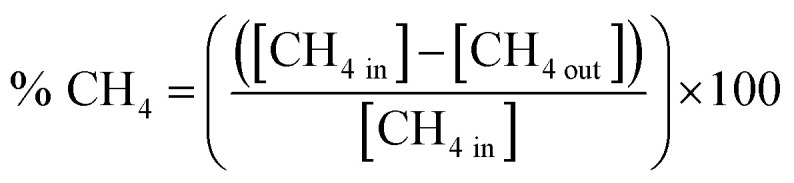
5
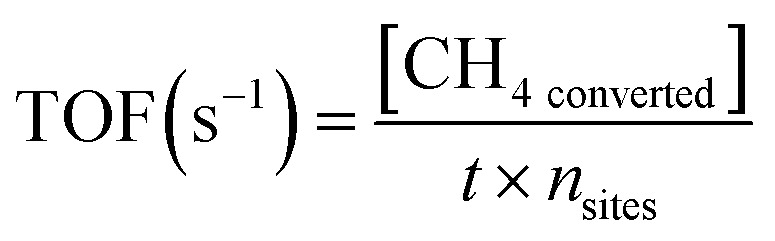
where s is seconds, *n* is the number of moles, [CH_4 converted_] is the amount of methane converted (g per cat per s) at a specific time (s), and *n*_sites_ is the number of active catalytic sites (g per cat).

### Characterization of materials

Several methods were used to characterize the as-prepared nanocatalysts. Their morphology was investigated using transmission electron microscopy (FEI Tecnai G2 and TF20 TEM) and scanning electron microscopy (Nova Nano SEM). Their compositions were probed using elemental dispersive X-ray spectroscopy (EDX) and inductively coupled plasma optical emission spectroscopy (ICP-OES). X-ray photoelectron spectroscopy (Therma Scientific K-Alpha+, monochromated Al Kα, 1486.6 eV, equipped with a charge-neutralizing flood gun) was used to examine the surface chemical states of the nanocatalysts. X-ray diffraction (PANalytical EMPYREAN) was used to analyze their crystalline structure. Thermogravimetric analysis (TGA) was used to examine thermal stability.

## Results and discussion

**Fig. 1:**
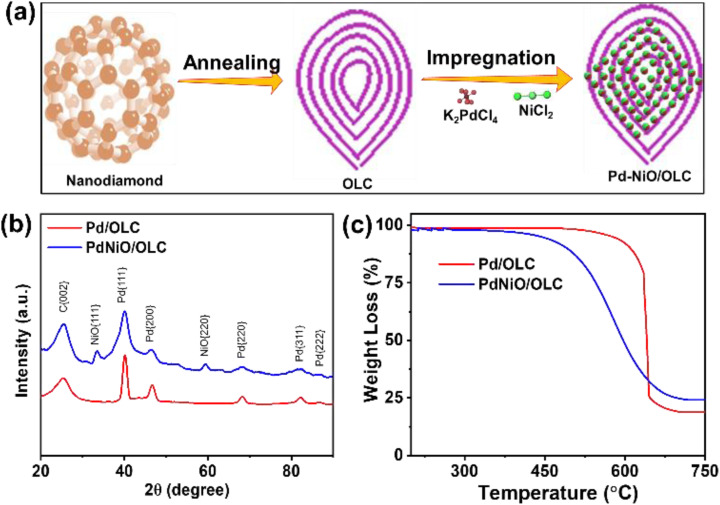
(a) Synthesis process, (b) XRD, and (c) TGA of Pd/OLC and PdNiO/OLC.

The OLC was prepared by thermally annealing the nanodiamond precursor, and successful immobilization of PdNiO and Pd nanoparticles was then achieved by impregnation on the OLC support (Fig. 1a). This was confirmed by XRD examination. The diffraction pattern of Pd/OLC shows distinct reflections at 2*θ* ≈ 40.03°, 46.62°, 68.25°, and 82.14°, which correspond to the (111), (200), (220), (311), and (222) crystallographic planes of face-centered cubic (fcc) Pd respectively, as well as the distinctive (002) reflection of graphitic carbon at 25.3° (Fig. 1b). However, there are slight positive shifts in the Pd peaks of PdNiO/OLC, implying lattice contraction upon the addition of NiO. The presence of NiO in the Pd/OLC catalytic matrix was confirmed by the additional diffraction peaks found in the PdNiO/OLC composite at 33.43° and 59.47°, which were attributed to the (111) and (220) planes of NiO.^10^ It is well known that adding NiO improves the efficacy of methane oxidation by producing more oxygen species and boosting surface oxygen mobility.^14^ Additionally, at every stage of the process, NiO reduces the competitive adsorption of hydroxyl and water species while promoting more uniform dispersion of nanoparticles, which increases the exposure of catalytically active sites.^14^

TGA-air investigation highlights the high-temperature durability of the Pd/OLC and PdNiO/OLC catalysts and confirmed their suitability for use in thermally demanding processes such as methane oxidation (Fig. 1c). The physically graphitic construction of the OLC support, which successfully reduces thermal deterioration, is responsible for the notable thermal stability. The Pd/OLC catalyst showed very little mass loss below 500 °C, suggesting its structural integrity in this temperature range. After little weight loss between 500 and 600 °C, it was followed by rapid carbon oxidation above 600 °C and full combustion at about 700 °C, metallic Pd was left as the residue. Conversely, PdNiO/OLC showed stability up to 400 °C, followed by modest degradation between 400 and 500 °C. Above 500 °C, however, there was a noticeable weight loss due to the improved carbon oxidation kinetics. The process came to an end near 700 °C, leaving behind the metallic composite (PdNiO). The significantly reduced thermal stability of PdNiO/OLC in comparison to Pd/OLC can be explained by the facilitation of oxygen transfer to the carbon matrix caused by the presence of NiO and related oxygen species.

**Fig. 2:**
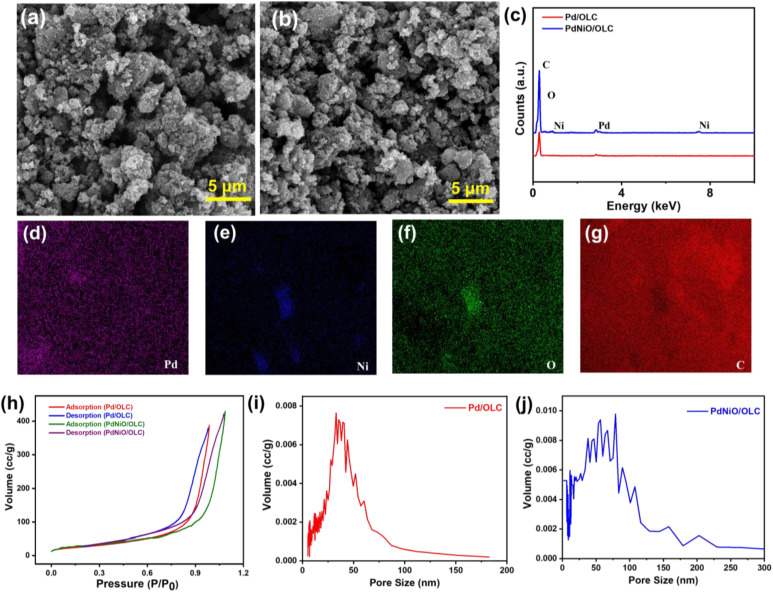
(a and b) SEM images, (c) EDX analysis, (d–g) elemental mapping of PdNiO/OLC, and (h–j) N_2_ adsorption–desorption isotherm and pore size distribution of Pd/OLC and PdNiO/OLC, respectively.

SEM micrographs of the Pd/OLC and PdNiO/OLC show sponge-like surface morphologies that are typical of highly porous materials (Fig. 2a and b). It offers a highly accessible surface area,^15^ facilitates effective mass transport,^16^ and improves structural stability and reusability over multiple catalytic cycles;^17^ these properties are beneficial for catalytic processes. With a total metal loading of about 20 wt%, the elemental composition ascertained by EDX verified the presence of carbon (C), oxygen (O), palladium (Pd), and nickel (Ni) (Fig. 2c). In addition, quantitative investigation of the PdNiO/OLC by ICP-OES indicates a Pd-to-Ni atomic ratio of 10.27 : 11.79, which is in line with the desired stoichiometry. Elemental mapping confirms the dispersion of mixed oxide nanoparticles (Pd, Ni, O) on the OLC support (C) in PdNiO/OLC (Fig. 2d–g). The mapping results reveal that Pd, O and C exhibit intense and continuous signals, while Ni shows concentric and sparse spots. This observation further corroborates an overlap of the Pd–O–Ni (Pd-NiO) phase uniformly spread on the OLC support. Interestingly, there are no discernible morphological differences between the Pd/OLC and PdNiO/OLC catalysts, indicating that the overall textural features seen in the SEM examination were not significantly changed by the addition of NiO.

According to the IUPAC classification system, the nitrogen (N_2_) adsorption–desorption isotherms of Pd/OLC and PdNiO/OLC show typical type-IV features (Fig. 2h), which are indicative of mesoporous materials.^18,19^ A homogeneous distribution of mesopores is indicated by the H1-type hysteresis loops seen in both catalysts.^18,19^ The structural contribution of the OLC framework to the improved textural qualities is reflected in the measured BET surface areas of 75 m^2^/g for Pd/OLC and 97 m^2^/g for PdNiO/OLC. The BJH pore volumes rise proportionally from 0.3 cm^3^/g for Pd/OLC and 0.6 cm^3^/g for PdNiO/OLC. Because of the partial surface remodelling brought about by NiO incorporation, which probably encourages increased pore accessibility and active site exposure, PdNiO/OLC has larger pore dimensions.^20^ This observation is further confirmed by the pore size distribution, with PdNiO/OLC having an increased mean pore size (54.08 ± 74.57 nm) compared to Pd/OLC (37.53 ± 42.21 nm) (Fig. 2i and j). The larger standard deviation than the mean indicates substantial variability of the data with some outliers.

**Fig. 3:**
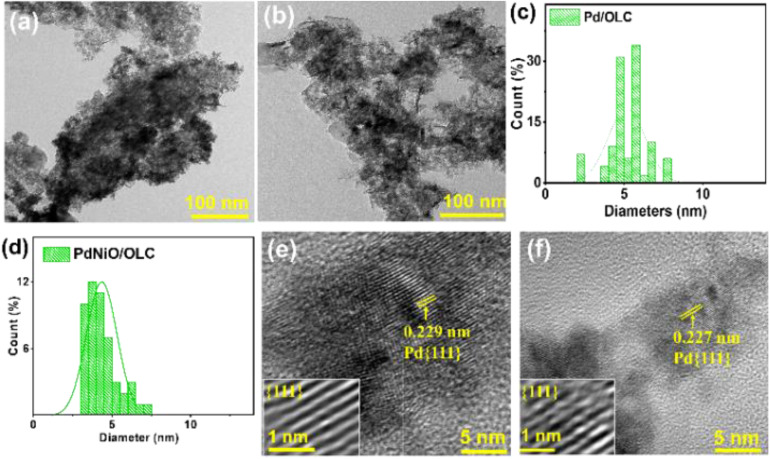
(a and b) TEM images, (c and d) particle size distribution, and (e and f) HR-TEM images of Pd/OLC and PdNiO/OLC.

It is evident from the TEM micrographs that the OLC support is covered in uniformly distributed nanocrystals for both the Pd/OLC and PdNiO/OLC catalysts (Fig. 3a and b). The average particle size of PdNiO/OLC is smaller at 4.3 ± 0.97 nm compared to 5.3 ± 1.12 nm for Pd/OLC (Fig. 3c and d). This slight decrease in the nanoparticle size of PdNiO/OLC further confirms the contractive effect of NiO in the lattice of Pd/OLC. This result corroborates the findings from XRD analysis. It was anticipated that the reduced nanoparticle size would enhance the metal use efficiency and increase the exposure of catalytically active areas.

Both catalysts exhibit distinct lattice fringes, as well as localized crystalline flaws such as stacking faults, interfacial, and intragranular dislocations in the high-resolution TEM examination (Fig. 3e and f). A feature that is frequently linked to Pd-based catalysts, non-epitaxial crystal formation, is suggested by the diversity in fringe orientation.^8^ Thermal methane oxidation may benefit from the additional active sites that these structural imperfections can produce, which are advantageous for surface reactions. For Pd/OLC, the lattice spacing corresponding to the Pd {111} plane was 0.229 nm, while for PdNiO/OLC, it dropped to 0.227 nm. The slight reduction in the fringe spacing of Pd in PdNiO/OLC is due to greater metal–support interaction and the synergistic electronic coupling between Pd and NiO.^21^ These factors work together to stabilize smaller particles and enhance catalytic efficiency.

**Fig. 4:**
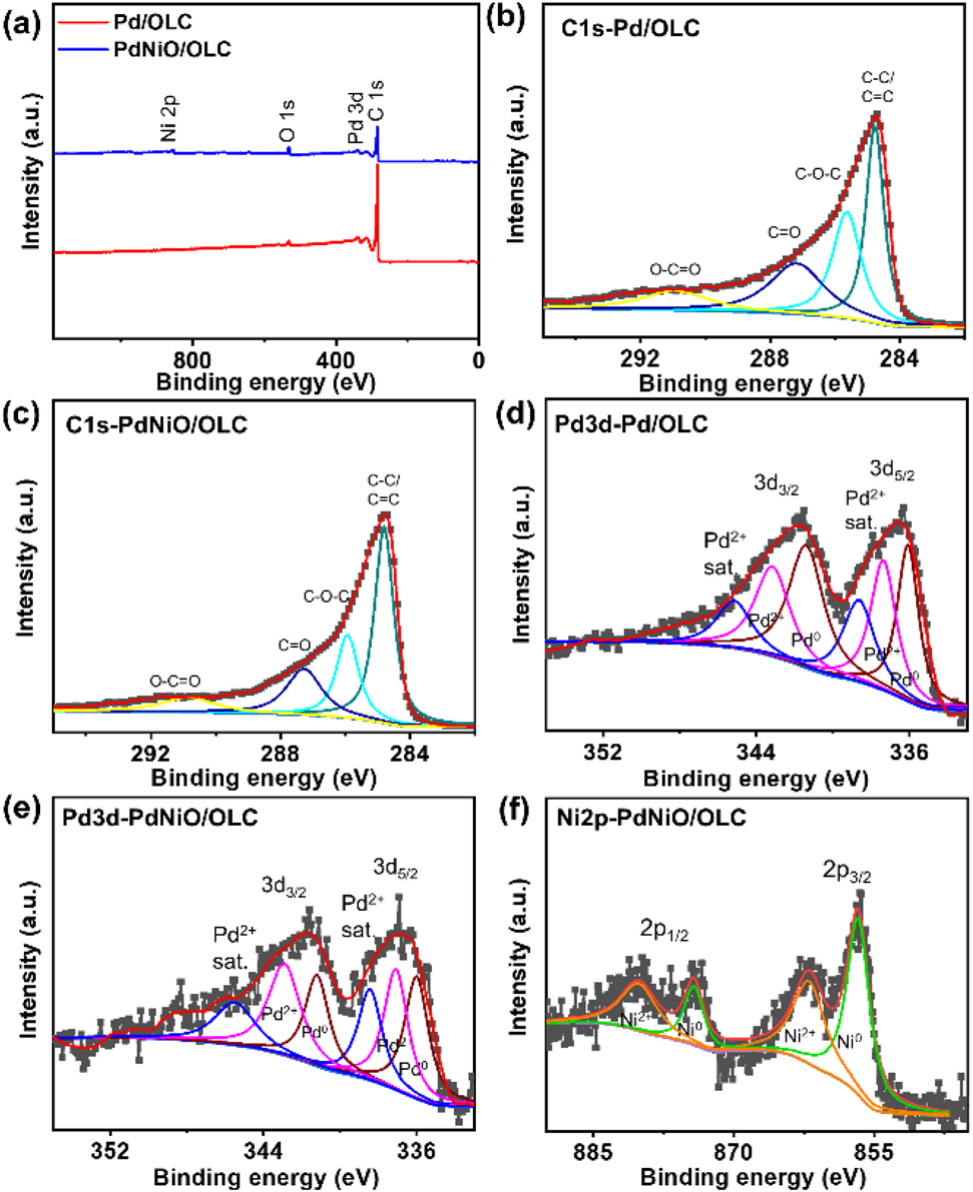
(a) XPS full surface survey and high-resolution (b and c) C 1s, (d and e) Pd 3d, (f) Ni 2p spectra of Pd/OLC and PdNiO/OLC, respectively.

The co-existence of the elements Pd, Ni, O, and C was confirmed from the XPS survey spectra of Pd/OLC and PdNiO/OLC catalysts (Fig. 4a). The partially oxidized character of the OLC support was confirmed by the C 1s spectra, which were dominated by peaks attributed to C–C and C=C bonds, with minor components due to C–O–C, C=O, and O–C=O surface functionalities (Fig. 4b and c). The Pd 3d spectra of both catalysts show distinctive doublets linked to metallic Pd^0^ as the dominating species, with a small contribution from the Pd^2+^ states (Fig. 4d and e).^22^ Compared to that of Pd/OLC, a slight decrease in the Pd 3d binding energy was noted for PdNiO/OLC, indicating electron redistribution at the Pd-NiO–OLC interface.^10^ This change is compatible with increased electronic coupling between Pd and Ni species and is suggestive of a higher Pd d-band center. The Ni (2p_3/2_ and 2p_1/2_) spectra of the PdNiO/OLC sample reveal a main peak typical of Ni^0^ in Ni 3p, along with a weak signal and satellite structure attributed to Ni^2+^ species (Fig. 4f). This is confirmed by the more significant metallic Pd and Ni phases compared to their oxide forms. The presence of oxides of the metals might be due to possible electronic interaction between Pd and NiO (*i.e.*, Pd–O–Ni).

**Fig. 5:**
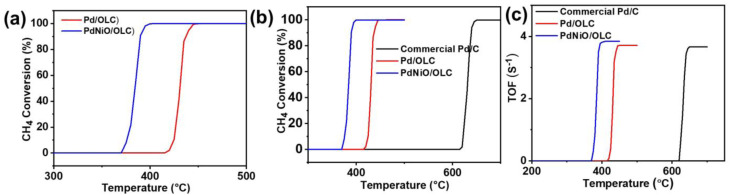
(a) Methane oxidation catalytic performance of Pd/OLC and PdNiO/OLC. (b) Comparison of the catalytic activity and (c) the TOF of all catalysts.

The catalytic methane oxidation activity was measured in a fixed-bed reactor between 25 and 650 °C. Active metal sites are necessary for methane activation, as demonstrated by the lack of detectable activity of the metal-free OLC support at all tested temperatures. Conversely, Pd/OLC showed notable catalytic activity, with complete methane conversion (*T*_100_) at 450 °C and a methane conversion onset temperature (*T*_onset_) of 415 °C (Fig. 5a). NiO was added to improve the dispersion of metal nanoparticles and increase the surface area, which further improved the catalytic reaction. Thus, full methane conversion was accomplished by PdNiO/OLC at a significantly lower *T*_onset_ (370 °C) and *T*_100_ (400 °C). The commercial Pd/C catalyst performed worse than both OLC-supported systems, as evidenced by the significantly higher activity thresholds (*T*_onset_ = 615 °C and *T*_100_ = 650 °C) (Fig. 5b). Because of the synergistic interactions among NiO, Pd, and the OLC support, which together enrich the surface with oxygen-containing species and encourage the thermocatalytic oxidation of methane,^14^ PdNiO/OLC exhibits superior methane oxidation activity. Additional information regarding intrinsic catalytic efficiency was obtained through the turnover frequency (TOF) determined using eqn (5). The highest TOF values were recorded for PdNiO/OLC at all tested temperatures, highlighting its higher intrinsic activity. Pd/OLC outperformed the commercial Pd/C catalyst by a large margin, although it had lower TOF values than PdNiO/OLC. This highlights the crucial role that metal dispersion and support composition play in catalytic performance (Fig. 5c).

**Fig. 6:**
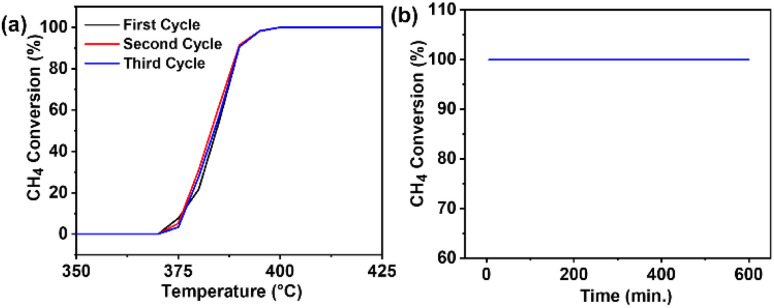
(a) Recycling tests and (b) stability test at 400 °C of PdNiO/OLC.

Out of all the materials examined, the as-prepared PdNiO/OLC catalyst had the best catalytic activity toward methane oxidation. Three consecutive catalytic cycles were conducted to verify its reusability. The PdNiO/OLC catalyst maintained the same *T*_onset_ (∼370 °C) and *T*_100_ (∼400 °C) values (Fig. 6a), exhibiting exceptional stability and recyclability. A time on stream (TOS) of 10 h has been considered appropriate for laboratory-scale methane oxidation in the literature.^23,24^ Hence, a continuous stability test at 400 °C for 10 h was used to confirm the long-term durability of PdNiO/OLC, and demonstrated full methane conversion without any signs of deactivation (Fig. 6b). This was corroborated by ICP-OES analysis of the spent catalyst after the reaction, which shows a similar Pd : Ni atomic ratio (10.31 : 11.83) to the as-prepared catalyst (10.27 : 11.79). This confirms that no metal leaching of PdNiO/OLC occurred after three consecutive reaction cycles. While PdNiO/OLC surpasses PdCe/MFI (425 °C), PdNi/Al_2_O_3_ (450 °C), and several other benchmark catalysts, it has similar performance to other previously published catalysts, such as PdNi/halloysite (402 °C),^25^ Pd@silicalite-1 (400 °C),^26^ Pt/CeO_2_ (400 °C),^27^ and PdZn/Al_2_O_3_ (400 °C)^28^ systems in terms of catalytic efficiency (Table 1). The positive interaction between Pd and NiO nanoparticles is responsible for the increased capacity to oxidize methane. By facilitating uniform Pd dispersion and encouraging the development of Pd-NiO interfacial sites, the incorporation of NiO lowers the methane activation energy and improves the reducibility of PdO. Additionally, PdNiO/OLC shows exceptional resistance to deactivation, in contrast to Pd/OLC. This is traced to the tendency of Pd active sites to facilitate C–H bond activation *via* chemisorbed oxygen species being improved by NiO interactions, which promote rapid reaction of the methyl radical and lattice oxygen to form carbon dioxide (CO_2_) and water (H_2_O) (eqn (6)–(12)). The mechanism is based on the redox cycle of Pd^0^ and Pd^2+^, enabled by the oxygen mobility arising from NiO interactions, thereby stabilizing the active PdO phases and promoting robust oxygen mobility for efficient methane oxidation. The strong metal–metal oxide interaction in PdNiO/OLC is essential to preserve the active surface sites and guarantee continuous catalytic activity during methane oxidation. The mechanistic reactions of methane oxidation on PdNiO/OLC are presented in detail below.

**Table 1:** Comparison of recent reported catalysts for methane oxidation

Catalyst	Support	Gas mixture (CH_4_ : O_2_)	*T*_100_ (°C)	Ref.
PdNi	Halloysite	1 : 20	402	25
Pd	Silicalite-1	1 : 4	400	26
Pd	Al_2_O_3_	1 : 90	∼550	29
Pt	CeO_2_	0.1 : 4	400	27
Pd	Al_2_O_3_	10 : 100	500	30
PdCo_3_O_4_	Al_2_O_3_	10 : 100	400	30
Pd	Al_2_O_3_	0.2 : 5	500	31
Pd	MFI	0.2 : 5	450	31
PdCe	MFI	0.2 : 5	425	31
Pd	CeO_2_	0.5 : 2.5	450	32
Pd	CeO_2_	0.2 : 20	450	33
PdNi	Al_2_O_3_	1 : 20	450	28
PdZn	Al_2_O_3_	1 : 20	400	28
Pd	OLC	1 : 20	450	This work
PdNiO	OLC	1 : 20	400	This work

❖ Adsorption/activation of methane on PdO active sites:6
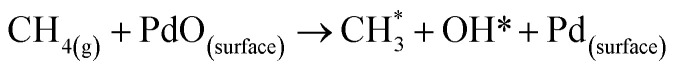


❖ Stepwise dehydrogenation of adsorbed methane intermediates:7
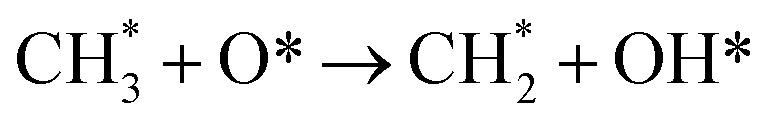
8
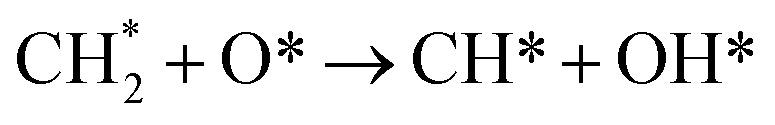

9 CH* + O* → C* + OH*

❖ Oxidation of carbon intermediates to CO:

10 C* + O* → CO*

❖ Oxidation of adsorbed CO to CO_2_ and desorption:

11 CO* + O* → CO_2(g)_ + PdO_(surface)_

❖ Water formation and desorption:

12 2OH* → H_2_O_(g)_ + O*

## Conclusion

The study shows that onion-like carbon (OLC) is a strong support for Pd and PdNiO nanocatalysts, greatly improving their catalytic and structural characteristics. Its extraordinary activity in methane oxidation catalysis was supported by the high surface area of the PdNiO/OLC composite (97 m^2^/g) and its superior thermal stability. According to XRD and XPS investigations, the addition of NiO to the Pd/OLC framework aided in the oxidation process by encouraging the production of oxygen-containing species. A uniform distribution of nanoparticles, smaller particle sizes, and a higher density of active sites were all observed *via* TEM. PdNiO/OLC achieved total methane conversion (*T*_100_) at 400 °C, which is significantly lower than those of Pd/OLC (450 °C), commercial Pd/C (650 °C), and the most recently reported catalysts in the literature. These physicochemical merits endowed PdNiO/OLC with exceptional catalytic efficiency. Additionally, the TOF of PdNiO/OLC greatly exceeded those of the comparative samples. The findings show that OLC is a flexible and promising support platform for thermocatalytic uses other than methane oxidation.

## Conflicts of interest

The authors declare no conflicts of interest.

## Data Availability

The data supporting this article have been included within the texts.
